# JMJD6–BRD4 complex stimulates lncRNA *HOTAIR* transcription by binding to the promoter region of *HOTAIR* and induces radioresistance in liver cancer stem cells

**DOI:** 10.1186/s12967-023-04394-y

**Published:** 2023-10-25

**Authors:** Ruifeng Pei, Le Zhao, Yiren Ding, Zhan Su, Deqiang Li, Shuo Zhu, Lu Xu, Wei Zhao, Wuyuan Zhou

**Affiliations:** Department of Hepatopancreatobiliary Surgery, Xuzhou Cancer Hospital, Gulou District, No. 131, Huancheng Road, Xuzhou, 221005 Jiangsu People’s Republic of China

**Keywords:** Liver cancer, Long noncoding RNA *HOTAIR*, JMJD6, LSD1, ERK2 (*MAPK1*), CD13+CD133+, JMJD6 inhibitor SKLB325, Radioresistance, Liver cancer stem cells

## Abstract

**Background:**

Long non-coding RNA (lncRNA) *HOTAIR* acts importantly in liver cancer development, but its effect on radioresistance remains poorly understood. Here, our study probed into the possible impact of *HOTAIR* in radioresistance in liver cancer stem cells (LCSCs) and to elucidate its molecular basis.

**Methods:**

Following sorting of stem and non-stem liver cancer cells, LCSCs were identified and subjected to RNA-seq analysis for selecting differentially expressed genes. Expression of *HOTAIR* was determined in liver cancer tissues and CSCs. The stemness, proliferation, apoptosis and radioresistance of LCSCs were then detected in response to altered expression of *HOTAIR*-*LSD1*-*JMJD6*-*BRD4*.

**Results:**

Ectopic *HOTAIR* expression was found to promote radioresistance of LCSCs by maintaining its stemness. Mechanistic investigations indicated that *HOTAIR* recruited *LSD1* to the *MAPK1* promoter region and reduced the level of H3K9me2 in the promoter region, thus elevating ERK2 (*MAPK1*) expression. JMJD6–BRD4 complex promoted *HOTAIR* transcription by forming a complex and positively regulated ERK2 (*MAPK1*) expression, maintaining the stemness of LCSCs, and ultimately promoting their radioresistance in vitro and in vivo.

**Conclusion:**

Collectively, our work highlights the promoting effect of the JMJD6–BRD4 complex on the radioresistance of LCSCs through a *HOTAIR*-dependent mechanism.

**Supplementary Information:**

The online version contains supplementary material available at 10.1186/s12967-023-04394-y.

## Introduction

Globally, the incidence of liver cancer continues to rapidly rise, and which has become the second leading reason contributing to cancer-related deaths [1–3]. Currently, surgical excision, liver transplantation and interventional radiological treatment are the most effective therapies for the treatment of low-grade liver cancer [4, 5]. However, because of indistinguishable or delayed appearance of clinical symptoms, few patients could receive above-mentioned managements [6]. Notably, the development of radiotherapy techniques has shown satisfied local–regional control effect for tumor cells, offering a promising option for most patients with advanced liver cancer [7]. But radio-resistance acquisition remains a serious obstacle for the successful treatment of liver cancer patients [8]. Thus, there is an urgent need to explore potential targets for overcoming radiotherapy resistance and further improving radiotherapy efficacy in the clinical settings.

Accumulating studies have demonstrated that cancer stem cells (CSCs), a distinct sub-population of cells, exhibit unlimited self-renewal potential, exclusive tumorigenicity, and resistance to chemo/radiotherapy [9–11]. LCSCs in liver cancer tissues may lead to resistance and metastasis, while standard managements cannot eradicate the resistant LCSCs [12, 13]. Hence, it is essential to reveal the underlying molecular mechanisms linked to LCSCs for the diagnosis and treatment of liver cancer [14]. CD13 and CD133 are generally recognized surface markers of LCSCs [15, 16], and based on the analysis of differentially expressed genes of CD13 and CD133-positive cell subgroups, we found that long non-coding RNA (lncRNA) HOX transcript antisense RNA (*HOTAIR*) is up-regulated in LCSCs. LncRNAs are non-coding RNAs consisting of more than 200 nucleotides in length [17]. LncRNAs compete with microRNAs (miRNAs) for binding and can be processed into miRNAs [18], and specifically, lncRNAs are implicated in diverse biological activities and bears great responsibility for modulation of diseases and cancers [19, 20]. *HOTAIR*, located in the Homeobox C gene cluster, is a lncRNA with regulatory functions of transcription and has been demonstrated to mediate gene expression through epigenetic modifications, like chromatin modifications [21] and DNA methylation [22, 23]. Multiple studies have indicated that overexpression of *HOTAIR* in liver cancer is positively correlated to poor prognosis, recurrence and tumor progression [24, 25]. A recent study has shown that *HOTAIR* regulates myeloid differentiation through the upregulation of p21 via miR-17-5p in acute myeloid leukaemia [26]. In addition, HOTAIR engages in myocardial regeneration in neonatal mice [27] and HOTAIR is capable of facilitating the activation and fibrosis of stellate cells in the liver [28]. Moreover, HOTAIR can regulate the development of leukemia by modulation of DNA and histone methylation [29]. Furthermore, *HOTAIR* has been highlighted to interact with lysine-specific demethylase 1 (*LSD1*) [30], and *LSD1* expression is enhanced in liver cancer cells [31]. Therefore, in the nucleus, *HOTAIR* tethers protein complexes to chromatin to repress or activate gene transcription, thus controlling the progression of liver cancer.

In addition, it is interesting to know that jumonji domain containing 6 (JMJD6) and bromodomain-containing protein 4 (BRD4) can be recruited to the *HOTAIR* promoter region to promote *HOTAIR* expression in breast cancer and glioblastoma multiforme [32, 33], and the JMJD6–BRD4 complex can modulate the expression of genes [34]. Furthermore, elevated expression of *JMJD6* can maintain cancer stemness properties [35], and SKLB325, an inhibitor of JMJD6, can effectively impede the progression of cancer [36]. Herein, from the above-mentioned findings, we hypothesize that the JMJD6–BRD4 complex may mediate *HOTAIR* expression, which in turn affects the radioresistance of liver cancer by interacting with *LSD1*. To address this hypothesis, we studied the correlation between JMJD6–BRD4 complex and *HOTAIR* expression in clinical tissue samples of liver cancer patients, LCSCs and xenograft tumor in nude mice, as well as the effect of their interactions on the radioresistance of LCSCs, aiming at clarifying the mechanistic actions of HOTAIR and providing a novel therapeutic target for liver cancer.

## Materials and methods

### Ethics statement

The clinical research was approved by the Ethics Committee of Xuzhou Cancer Hospital and conducted in accordance with the *Declaration of Helsinki*. All participants signed informed consent documentation before sample collection.

### Study subjects

This study enrolled 50 patients (aged 31 to 68 years) diagnosed with liver cancer by aspiration biopsy and pathological examination at Xuzhou Cancer Hospital from September 2017 to February 2019. The diagnosis of liver cancer was carried out according to the standards of the World Health Organization. All the tumor samples were confirmed to contain more than 80% tumor cells through pathological examination. Tissue samples were obtained from patients who did not receive any anti-cancer treatment. Pathological examination was performed to confirm that all tumor nodules were completely resected by removing tumor-free tissue on the liver surface. Complete clinical pathology and follow-up data were collected, and the death cases from non-liver cancer or accidents were excluded.

### Immunohistochemistry

Paraffin-embedded and sectioned clinical tissue specimens were dewaxed to water, dehydrated in an alcohol gradient, washed twice in distilled water, treated with 3% methanolic hydrogen peroxide for 20 min, washed in distilled water for 2 min, and 3 min in 0.1 M PBS. Then, the tissue specimens were subjected to antigen retrieval in a water bath and cooled in tap water. Afterwards, the tissue specimens were exposed to normal goat serum solution (Shanghai Haoran Biotechnology, Shanghai, China) at room temperature for 20 min and incubated with primary antibody [rabbit anti-human JMJD6 (1:500, ab256798, Abcam, Cambridge, UK), LSD1 (1:200, ab129195, Abcam), ERK2 (*MAPK1*) (1:200, 9926, CST), Ki-67 (1:250, ab15580, Abcam)] at 4 °C overnight. Next, the tissue specimens were washed three times with 0.1 M PBS and then treated with goat anti-rabbit immunoglobulin G (IgG) (1:500, ab6785, Abcam) as secondary antibody at 37 °C for 20 min. Further, the tissue specimens were incubated with horseradish peroxidase (HRP)-labeled streptavidin protein working solution (0343-10000U, Yimo Biotechnology, Beijing, China) at 37 °C for 20 min, and then respectively stained with 3,3ʹ-diaminobenzidine tetrahydrochloride (DAB) (ST033, Guangzhou WHIGA biotechnology, Guangzhou, Guangdong, China) and hematoxylin (PT001, Shanghai Bogoo Biotechnology, Shanghai, China). Five fields with high magnification were randomly selected under microscope (Axio Vert. A1, Zeiss, Germany) for each slice to calculate the positive rate of immunohistochemistry.

### Cell culture

Human liver cancer cells (Hep3B and Huh7) and human embryonic kidney cells 293T were purchased from Wuhan Procell Life Science & Technology Co., Ltd. (Wuhan, China). Cells were cultured in Dulbecco’s modified Eagle’s medium (DMEM; Gibco, Grand Island, NY) containing 10% fetal bovine serum (FBS; Gibco), 100 μg/mL streptomycin and 100 U/mL penicillin in a 5% CO_2_ incubator (Thermo Fisher Scientific Inc., Waltham, MA) at 37 °C. Upon reaching about 75% confluence, the cells were seeded for experiments.

### CD13^+^ and CD133^+^ cell sorting

Normally cultured Hep3B and Huh7 cells were routinely digested to prepare single cell suspension, and then the cells were collected after centrifugation at 160*g* for 5 min. Afterward, above-mentioned cells were incubated with fluorescein isothiocyanate-conjugated CD13 (1:500, cat#11-0138, eBioscience, San Diego, CA) and phycoerythrin-conjugated CD133 (1:300, cat#130-098-826, Miltenyi Biotec, Auburn, CA) alone or in combination at 4 °C for 30 min. Following this, the sample was obtained by centrifugation at 160*g* for 5 min and sorted by a flow cytometer (BD FACSVerse, USA). The sorted cells were cultured with DMEM (Sigma-Aldrich Chemical Company, St Louis, MO) containing 10% FBS (HyClone Laboratories, Logan, Utah).

### Plasmids, shRNAs and cell transduction

First, 293T cells were transfected with Lipofectamine 2000 reagent (Invitrogen), and then the supernatant with virus particles was collected to infect recipient cells (CD13+CD133+ Hep3B and Huh7 cells). After 48 h of transfection, the transfected positive cells were selected for follow-up experiments. The cells were transduced with shRNA control (shCtl), shRNA targeting *HOTAIR* (sh*HOTAIR*), shRNA targeting *JMJD6* (sh*JMJD6*)#1, sh*JMJD6*#2, sh*LSD1*#1, sh*LSD1*#2, overexpression plasmid negative control (oe-NC) (Plvx-vector), oe-ERK2 (*MAPK1*), oe-*LSD1* and oe-*HOTAIR*. LZRS-*HOTAIR* was obtained from Addgene Inc (Cambridge, MA) [37], while PLV-*ERK2* and PLV-*LSD1* from Yunzhou Biological Technology (Guangzhou) Co., Ltd. (Guangzhou, Guangdong, China) (pLV-*ERK2* (vector ID: VB900120-5363pxd). The used plasmid concentration of pLV-*LSD1* (vector ID: VB900000-0293ahx) was 50 ng/mL.

Radiosensitivity experiment: X-ray (6 Gy) was employed to irradiate cells subjected to shCtl, sh*HOTAIR*, sh*JMJD6* + shCtl, sh*LSD1* + shCtl, shCtl + oe-NC, sh*HOTAIR* + oe-NC, sh*HOTAIR* + oe-*LSD1*, DMSO treatment, and SKLB325 (4 μM, *JMJD6* inhibitor) treatment. The shRNA sequence is shown in Additional file 1: Table S1.

### Radiotherapy treatment

The Astrophysics Torrex X-ray Inspection System (Model 120D, Scanray Corporation) was used to irradiate cells with X-ray at 115 kVp and 5 mA. The cells in the logarithmic growth phase were seeded in DMEM containing 10% FBS, and 100 U/mL penicillin–streptomycin at 1 × 10^5^ cells/mL, and cultured in a 5% CO_2_ incubator at 37 °C. If transfection was needed, the cells were irradiated 24 h after transfection with 6 Gy (dose rate: 0.341 Gy/min, radiation area: 10 cm × 10 cm) of X-ray, and continued to be cultured for 24 h for subsequent experiments.

### Microsphere formation assay

The standard medium was made by serum-free DMEM/F12 (1:1) basic medium, the hepatocyte growth factor (HGF) (10 ng/mL), the recombinant growth factor (bEGF) (20 μg/mL) and B27. The cells to be tested were cultured with 2 mL standard medium at a low-adhesion six-well plate in the incubator (Thermo Fisher Scientific, Inc., Waltham, MA) with 37 °C and 5% CO_2_. Two weeks later, the images of cells were obtained under a microscope and the number of microspheres with volume greater than 100 μm was counted.

### Clonogenic assay

The cells to be tested were seeded into 6-well plates, and exposed to X-ray (6 Gy) upon reaching the density of approximately 50%. After 7 days, the culture medium was discarded, and the cells were fixed with methanol for 10 min. Next, the cells were stained with crystal violet, and the results were recorded by photos. More than 50 cell clones were counted under an ordinary inverted microscope, and then the colony formation rate was calculated as follows: ${\text{colony formation rate }}\left( \% \right)\, = \,\left( {{\text{number of colonies}}/{\text{number of inoculated cells}}} \right)\, \times \,{1}00\% .$

### Western blot

The extracted protein was separated by 10% sodium dodecyl sulfate–polyacrylamide gel electrophoresis and then electro-transferred to the polyvinylidene fluoride membrane. After blocking with 5% interaction of bovine at room temperature for 2 h, the membrane was incubated with diluted primary antibody [JMJD6 (1:1000, ab64575, Abcam), LSD1 (1:1200, ab17721, Abcam), ERK2 (*MAPK1*) (1:1000, 9926, CST) and BRD4 (1:1500, ab75898, Abcam)] at 4 °C overnight. HRP-conjugated corresponding secondary antibody (goat anti-rabbit proteintech SA00001-1; goat anti-mouse proteintech SA00001-2) was added to incubate with membrane at room temperature for 1 h. Further, the membrane was exposed to enhanced chemiluminescence (EMD Millipore Corporation, Billerica, MA) and immunoblots were visualized and captured by Bio-Rad imaging system. Glyceraldehyde-3-phosphate dehydrogenase (GAPDH) (1:1000, ab181602, Abcam) was used as an internal reference, and the protein band image was analyzed by Image Lab software.

### Quantification of gene expression

RT-qPCR was adopted to measure the mRNA expression of target genes. According to the instructions, TRIzol kit (Invitrogen, Carlsbad, CA) was employed to extract total RNA from tissues or cells and measured RNA concentration. The primers used in this study were synthesized by Dalian Takara Biotechnology Co., Ltd. (Dalian, Liaoning, China) (Additional file 1: Table S2). Then RT-qPCR assay was conducted by complementary DNA reverse expression kit (K1622, Beijing Yaanda Biotechnology Co., Ltd., Beijing, China) and fluorescence quantitative PCR system (ViiA 7, Da An gene Co., Ltd. of Sun Yat-sen university, Guangzhou, Guangdong, China). The expression of genes was normalized to *GAPDH*, and the results were calculated by 2^−△△CT^ method.

### ChIP-PCR assay

ChIP-PCR assay was completed with antibodies [*JMJD6* (1:100, 16476-1-AP, Proteintech ProteinTech Group, Chicago, IL), LSD1 (1: 50, #2139, Cell Signaling Technology, Beverly, MA), H3K9me2 (1:100, ab1220, Abcam), *BRD4* (1:100, #83375, Cell Signaling Technology), and IgG (serving as a NC)]. The DNA fragments were recovered, and PCR amplification was performed to detect the binding of *JMJD6*/*BRD4* to the *HOTAIR* gene promoter region as well as the binding of *LSD1* and H3K9me2 in the *MAPK1* promoter.

### RIP assay

According to the manufacturer’s instructions, LSD1 antibody (1:50, #2139, Cell Signaling Technology), BRD4 (1:100, #83375, Cell Signaling Technology), and Magna RIP™ RIP kit (Millipore, Bedford, Massachusetts) were used for RIP assay. RNA was isolated from the immunoprecipitation product of the cells and quantified by a nanophotometer spectrophotometer (Implen Applications Note, Munich, Germany) of UV–visible light spectra. Enrichment of *HOTAIR* or LSD1 was detected by RT-qPCR.

### Cellular tumorigenicity in nude mice

Hep3B cells (6 × 10^6^ cells) were obtained and resuspended in 50 μL mixture (PBS: Matrigel = 1:1). Thirty nude mice, aged 5–6 weeks, were purchased from Shanghai SLAC Laboratory Animal Co., Ltd. (Shanghai China) and subcutaneously injected with above-mentioned cells. After 10 days, the injection site was treated with X-ray irradiation (10 Gy). These nude mice were equally divided into 5 groups (n = 6): shCtl + oe-NC group, sh*JMJD6* + oe-NC group, sh*JMJD6* + oe-*ERK2* group, PBS group and SKLB325 (*JMJD6* inhibitor) group. After tumor cell injection for 7 days, the mice were intraperitoneally injected with PBS or SKLB325 once a week for 4 consecutive weeks. Following this, the mice were euthanized, and the tumor tissues were excised, measured and photographed.

### TUNEL assay

The procedures before hydration were the same as the immunohistochemistry process. Next, slices were immersed in 0.85% NaCl and 4% paraformaldehyde in sequence, and then exposed to 20 μg/mL proteinase K solution. After treatment with 4% paraformaldehyde, the slices were incubated with rTdT reaction solution and then stained with 2× SSC solution. Thereafter, the slices were incubated with HRP and added with DAB for color reaction. Following sealing with neutral resin, the slices were observed under a microscope, with the positive rate calculated.

### RNA-seq analysis

Illumina NextSeqCN500 with Ribo-Zero protocol (Illumina, Inc., based in San Diego, CA) can be used to generate HiSeqLibrary with 950 ng mRNA. RNA sequencing (Illumina HiSeq) was performed on CD13^+^CD133^+^ LCSC subsets and negative liver cancer cell subsets. Next, Illumina's RTA (v1.18) software was used for image analysis and base detection. The sequencing library was generated using NEBNext® UltraTM RNA Library Prep Kit for Illumina® (NEB) according to the manufacturer’s recommendations, and the index code was added to the attribute sequence of each sample. Demultiplexing and FASTQ format file generation and alignment were performed using HiSeq analysis software (TopHat v2.1.1) and UCSC human genome version mm10. Thereafter, RNA-SeQC v1.1.8 was used for quality control, and HTSeq-counts v0.7.2 was used to generate counts. After converting the count data into FPKM (Fregments Per Kilobase per Million) format data, the R “limma” software package was applied to determine the differentially expressed genes between the CD13^+^CD133^+^ LCSC subsets and the negative liver cancer cell subsets with |logFC|> 1 and* p*.value < 0.05 as cut-off values.

### In silico analysis

For RNA-seq data, TCGA database was used to obtain the RNA-seq data in the FPKM format in the liver cancer project, which were then converted into the FPKM format into transcripts per million reads format and subjected to log2 conversion. The Wilcoxon rank sum test was adopted to calculate the difference in genes between the normal and tumor groups. A value of* p* < 0.05 was considered to be indicative of statistical significance. The clinical data of liver cancer patients were obtained from TCGA database. The “survminer” package (version 0.4.9) (for visualization) and “survival package” (version 3.2–10) (for statistical analysis of survival data) were employed to analyze the correlation between the expression of *LSD1* and ERK2 (*MAPK1*) and the overall survival of liver cancer patients. The expression grouping was set to 0–50 vs 50–100, and the statistical method was Log-rank test. A value of* p* < 0.05 was considered to be indicative of statistical significance.

### Statistical analysis

The statistical analyses were performed by SPSS 21.0 (IBM Corp., Armonk, NY), and the measurement data were expressed as mean ± standard deviation. Data obeying normal distribution and homogeneity of variance between cancer tissues and adjacent normal tissues were compared by paired *t* test, and those between the other two groups were compared by unpaired *t* test. The data comparison between multiple groups was performed by one-way analysis of variance (ANOVA) with Tukey's post-hoc test. Kaplan–Meier method was employed to evaluate the survival of patients, and log-rank test was used for univariate analysis. The results were considered statistically significant when *p* < 0.05.

## Results

### HOTAIR is upregulated in liver cancer tissues and LCSCs, which correlates to stemness maintenance and radioresistance of LCSCs

**Fig. 1:**
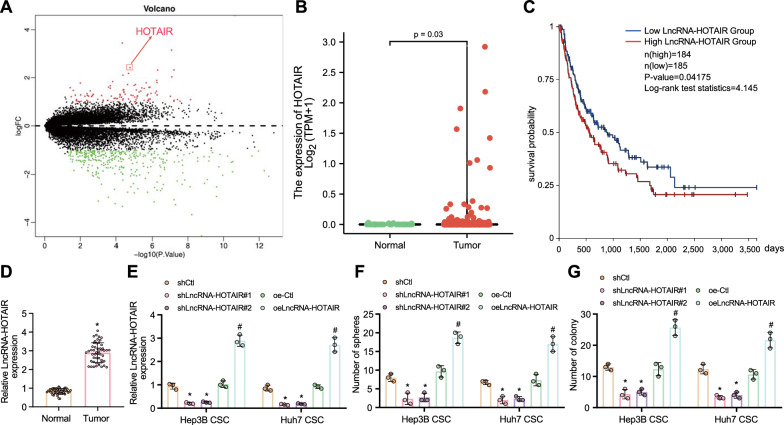
*HOTAIR* expression is increased in liver cancer tissues and LCSCs, which links to stemness maintenance and radioresistance of LCSCs. **A** A Volcano map of the gene expression between CD13+CD133+ liver cancer cell subsets and negative liver cancer cell subsets based on the RNA-seq data. Red indicates highly expressed genes while green indicates poorly expressed genes. **B** The expression of *HOTAIR* in liver cancer and normal tissue samples in TCGA database (*p* = 0.03). **C** Correlation between the expression of *HOTAIR* and the progression free survival of patients with liver cancer. **D** The expression of *HOTAIR* in normal and liver cancer tissues measured by RT-qPCR, normalized to *GAPDH*. *p < 0.05 compared with adjacent normal tissues. **E** Silencing and overexpression efficiency of *HOTAIR* determined by RT-qPCR in Hep3B and Huh7 CSCs. *p < 0.05 compared with Hep3B and Huh7 CSCs treated with shCtl, #* p* < 0.05 compared with Hep3B and Huh7 CSCs treated with oeCtrl. **F** The effect of *HOTAIR* silencing or overexpression on the stemness maintenance of LCSCs, as detected by microsphere formation assay. **G** The colony formation ability of LCSCs after 6 Gy X-ray irradiation after *HOTAIR* silencing or overexpression, as examined by clonogenic assay. *p < 0.05 compared with Hep3B and Huh7 CSCs treated with shCtl, #* p* < 0.05 compared with Hep3B and Huh7 CSCs treated with oeCtrl. Data were represented as mean ± standard deviation. Data between cancer tissues and adjacent normal tissues were compared by paired *t* test, and those between the other two groups were compared by unpaired *t* test. The data comparison between multiple groups was performed by one-way ANOVA with Tukey’s post-hoc test. Cellular experiments were repeated in triplicate

It has been recognized that CD13 and CD133 are surface markers of LCSCs [16]. Through flow cytometric sorting, the CD13^+^CD133^+^ liver cancer cell subsets in Hep3B and Huh7 cells were enriched and respectively defined as Hep3B and Huh7 CSCs. Analysis of differentially expressed genes in CD13^+^CD133^+^ or negative liver cancer cell subsets in the RNA-sequencing (RNA-seq) data suggested that *HOTAIR* was highly expressed in LCSCs, with significant difference (Fig. 1A). The differential gene heat map is shown in Additional file 2: Figure S1. In addition, the lncDiseas database showed that *HOTAIR* was related to hepatocellular carcinoma diseases (Additional file 1: Table S3). Furthermore, analysis using TCGA database indicated that *HOTAIR* was abundantly expressed in cancer tissues of patients with liver cancer (Fig. 1B) (*p* = 0.03) and that the expression of *HOTAIR* was negatively correlated with the progression free survival of patients with liver cancer (Fig. 1C). As illustrated by RT-qPCR, *HOTAIR* expression was higher in liver cancer tissues than that in adjacent normal tissues (Fig. 1D).

RT-qPCR results confirmed the silencing and overexpression efficiency of *HOTAIR* in Hep3B and Huh7 CSCs (Fig. 1E). The microsphere formation assay data displayed that *HOTAIR* silencing reduced the stemness of LCSCs, while overexpression of *HOTAIR* led to reversed condition (Fig. 1F, Additional file 2: Figure S2A). Meanwhile, *HOTAIR* silencing facilitated the inhibitory effect of X-ray irradiation on the colony formation ability of LCSCs, while opposite trend was witnessed upon overexpression of *HOTAIR* (Fig. 1G, Additional file 2: Figure S2B). The aforementioned results indicated that *HOTAIR* was overexpressed in liver cancer tissues and LCSCs. This overexpression was related to stemness maintenance and radioresistance of LCSCs.

### LSD1 interacts with HOTAIR to maintain the stemness of LCSCs and enhance their radioresistance

**Fig. 2:**
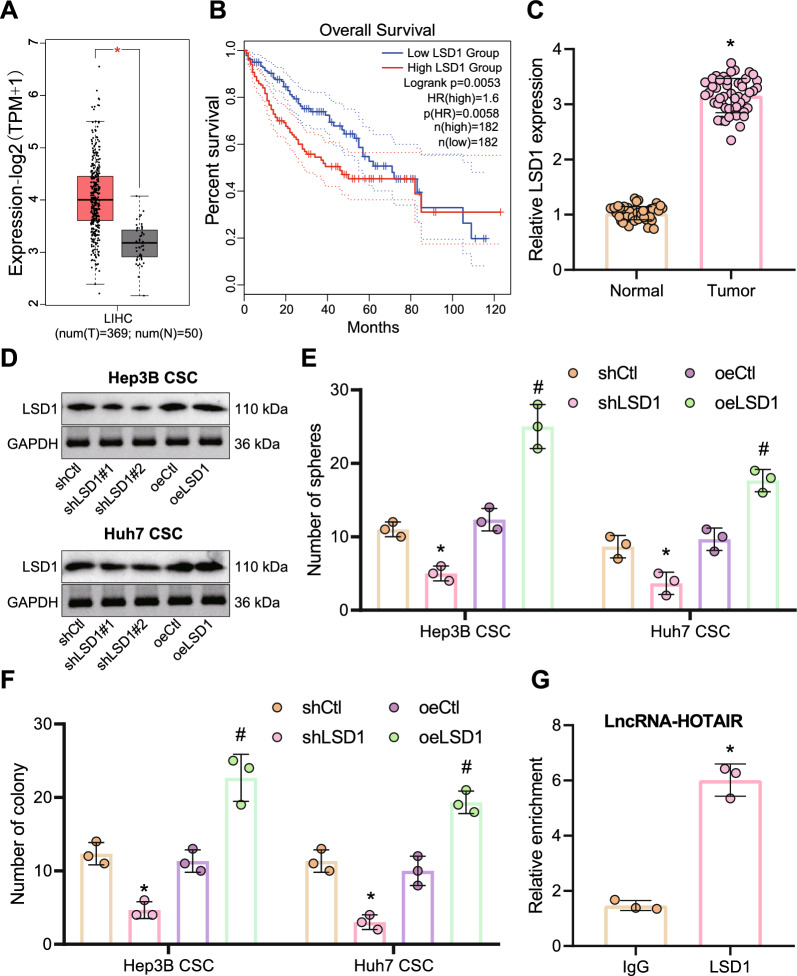
*LSD1* maintains the stemness of LCSCs and enhances cell radioresistance by interacting with *HOTAIR*. **A** The expression of *LSD1* in liver cancer tissues was analyzed based on TCGA database. LIHC, liver hepatocellular carcinoma. The red box plots represent tumor samples and the gray box plots represent normal samples. *p < 0.01 compared with normal samples. **B** Correlation between the expression of *LSD1* and the overall survival of patients with liver cancer. The red line indicates the liver cancer patients with high expression of *LSD1*, and the blue line indicates liver cancer patients with low expression of *LSD1*. The upper right is the significant *p* value and legend. **C** The expression of *LSD1* in adjacent normal tissues and liver cancer tissues, as measured by RT-qPCR, normalized to *GAPDH*. *p < 0.05 compared with adjacent normal tissues. **D** The silencing and overexpression efficiency of LSD1 was detected by Western blot, normalized to GAPDH. **E** The effect of *LSD1* depletion or overexpression on LCSCs stemness, as measured by microsphere formation assay. *p < 0.05 compared with shCtl-treated LCSCs, #*p* < 0.05 compared with oeCtl-treated LCSCs. **F** The colony formation ability of LCSCs with *LSD1* depletion or overexpression after treatment with 6 Gy X-ray irradiation, as examined by clonogenic assay. *p < 0.05 compared with shCtl-treated LCSCs, #*p* < 0.05 compared with oeCtl-treated LCSCs. **G** Interaction between *HOTAIR* and *LSD1* assessed by RIP assay. The relative enrichment on the Y-axis represents IP-*LSD1*/IgG. *p < 0.05 compared with the IgG group. The experimental data were summarized as mean ± standard deviation. Data between cancer tissues and adjacent normal tissues were compared by paired *t* test, and those between the other two groups were compared by unpaired *t* test. The data comparison between multiple groups was performed by one-way ANOVA with Tukey’s post-hoc test. Cellular experiments were repeated three times

*LSD1* has been reported to interact with *HOTAIR* in cancer cells [30], and the expression of *LSD1* is enhanced in liver cancer cells [31]. Similar to *HOTAIR*, *LSD1* was found to be overexpressed in clinical samples of patients with liver cancer, positively linked to the malignancy degree (Fig. 2A). In addition, *LSD1* expression was negatively linked to the overall survival of liver cancer patients (Fig. 2B). Moreover, *LSD1* expression was much higher in liver cancer tissues (Fig. 2C).

To analyze the role of *LSD1* in the stemness maintenance of LCSCs and their radioresistance, we silenced *LSD1* in Huh7 and Hep3B CSCs. The results of Western blot presented that both sh*LSD1*#1 and sh*LSD1*#2 sequences effectively decreased the expression of LSD1, with sh*LSD1*#2 showing the better silencing efficiency and was selected for following experimentations; and overexpression efficiency of LSD1 was also confirmed (Fig. 2D). The percentage of cells that formed microspheres was measured in Hep3B and Huh7 CSCs with *LSD1* depletion, and the results showed that *LSD1* depletion reduced the ability of LCSCs to form microspheres, while overexpression of LSD1 led to contrary finding (Fig. 2E, Additional file 2: Figure S2C), revealing critical role of *LSD1* in the maintenance of LCSCs. In addition, the colony formation ability of LCSCs was inhibited in response to *LSD1* silencing and X-ray exposure, while opposing tendency was detected upon overexpression of LSD1 (Fig. 2F, Additional file 2: Figure S2D). Furthermore, the results of RIP assay revealed that *HOTAIR* interacted with LSD1 (Fig. 2G). These results uncovered that *LSD1* could interact with *HOTAIR* and that *LSD1* maintained the stemness of LCSCs and thereby reduced the sensitivity of LCSCs to radiotherapy.

### HOTAIR promotes extracellular signal-regulated kinase 2 (ERK2) expression by recruiting LSD1 to the promoter region of MAPK1

**Fig. 3:**
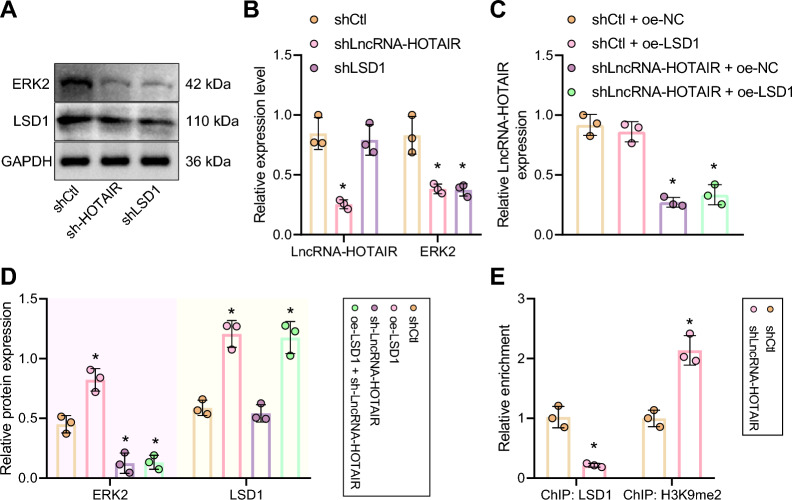
*HOTAIR* increases ERK2 (*MAPK1*) expression by recruiting *LSD1* to the promoter region of *MAPK1*. **A** ERK2 (*MAPK1*) and LSD1 expression in Hep3B and Huh7 CSCs with *LSD1* silencing or *HOTAIR* silencing as examined by Western blot, normalized to GAPDH. **B** *MAPK1* and *HOTAIR* expression in Hep3B and Huh7 CSCs with *LSD1* silencing or *HOTAIR* silencing examined by RT-qPCR, normalized to *GAPDH*. *p < 0.05 compared with Hep3B and Huh7 CSCs treated with shCtl. **C** The expression of *HOTAIR* in Hep3B CSCs treated with sh*HOTAIR* or combined with oe-*LSD1* measured by RT-qPCR, normalized to *GAPDH*. *p < 0.05 compared with shCtl + oe-NC-treated Hep3B CSCs. **D** Western blot assay of ERK2 (*MAPK1*) and LSD1 proteins in Hep3B CSCs treated with sh*HOTAIR* or combined with oe-*LSD1*, normalized to GAPDH. **E** The relative enrichment of *LSD1* and H3K9me2 in the promoter region of *MAPK1* in Hep3B CSCs with *HOTAIR* silencing assessed by ChIP, relative to Input. *p < 0.05 compared with shCtl-treated Hep3B CSCs. The experimental data were represented as mean ± standard deviation. Results between two groups were compared by unpaired *t* test. The data comparison between multiple groups was performed by one-way ANOVA with Tukey’s post-hoc test. Cellular experiments were repeated for three times

Previous reports have revealed that *HOTAIR* has been recognized as a scaffold molecule of *LSD1* to modulate the expression of genes [30, 38], and *LSD1* could further promote ERK expression [38]. More importantly, abnormal activation of ERK could enhance radioresistance of liver cancer cells [39]. Therefore, we hypothesized that *HOTAIR* might act as a scaffold molecule to recruit *LSD1* to the *MAPK1* promoter region, further mediate ERK2 (*MAPK1*) expression, and ultimately increase radioresistance of liver cancer. For validation, we first detected the expression of ERK2 (*MAPK1*) in Hep3B and Huh7 CSCs by RT-qPCR and Western blot, the results of which showed that the protein and mRNA levels of ERK2 (*MAPK1*) were diminished in Hep3B and Huh7 CSCs after *LSD1* depletion or *HOTAIR* depletion (Fig. 3A, B). Furthermore, *LSD1* overexpression could up-regulate ERK2 (*MAPK1*) expression, while simultaneous treatment of sh*HOTAIR* and overexpression (oe)-*LSD1* in Hep3B CSCs didn’t cause significant changes in the ERK2 (*MAPK1*) expression (Fig. 3C, D), implying that the effect of *LSD1* on ERK2 (*MAPK1*) depended on *HOTAIR*. Meanwhile, the results of ChIP assay suggested that in the presence of *HOTAIR* depletion, the binding of LSD1 to the *MAPK1* promoter region was attenuated and the H3K9me2 enrichment was increased (Fig. 3E). The above data demonstrated that *HOTAIR* could recruit *LSD1* to the promoter region of *MAPK1* and then trigger the demethylation of H3K9me2 in the *MAPK1* promoter, thus modulating ERK2 (*MAPK1*) expression.

### LSD1 enhances ERK2 (MAPK1) expression, thus maintaining the stemness of LCSCs and enhancing their radioresistance

**Fig. 4:**
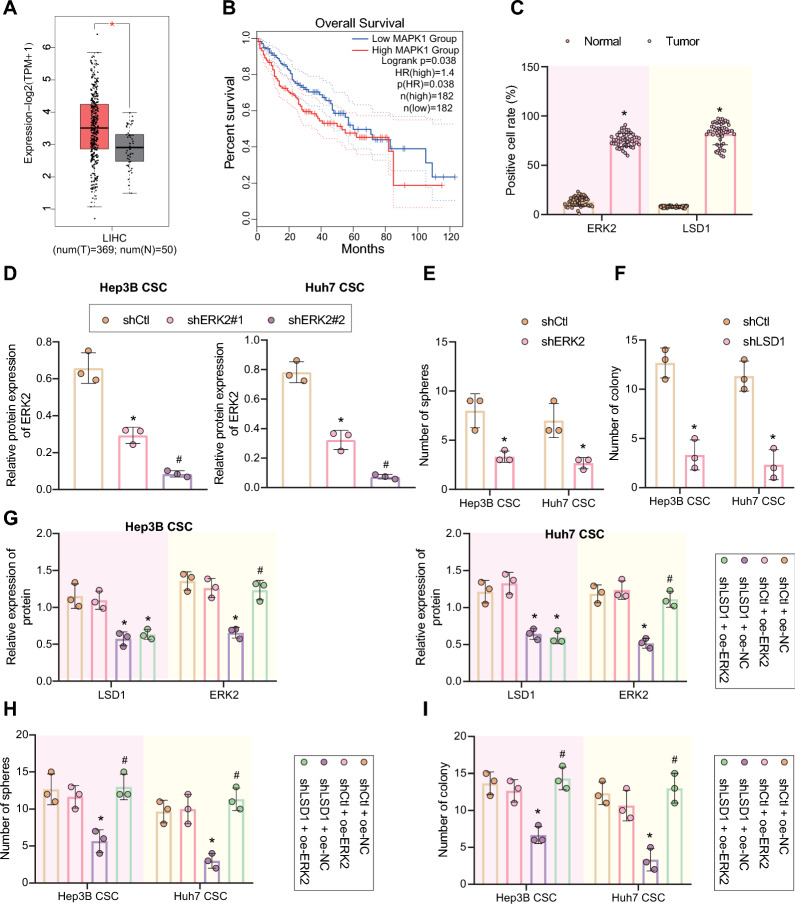
*LSD1* increased *ERK2 *(*MAPK1*) expression, and thus maintained LCSCs stemness and further enhanced their radioresistance. **A** *ERK2 *(*MAPK1*) expression in normal samples and liver cancer samples, as analyzed by TCGA database. LIHC, liver hepatocellular carcinoma. Red box plots represent tumor samples, and gray box plots represent normal samples. *p < 0.01 compared with normal samples. **B** The correlation between *ERK2 *(*MAPK1*) expression and overall survival rate of liver cancer patients analyzed by TCGA database. The upper right is the significant *p* value and legend. **C** The positive expression of *LSD1* and *ERK2 *(*MAPK1*) proteins in clinical liver cancer tissue samples, as measured by immunohistochemistry. *p < 0.05 compared with adjacent normal tissues. **D** The silencing efficiency of *ERK2 *(*MAPK1*) shRNAs in Hep3B and Huh7 CSCs, as detected by Western blot, normalized to *GAPDH*. **E** Microsphere formation assay was adopted to measure the stemness of LCSCs with *ERK2 *(*MAPK1*) silencing. *p < 0.05 compared with shCtl-treated cells. **F** The colony formation ability of LCSCs with *ERK2 *(*MAPK1*) silencing after exposure to 6 Gy X-ray, as measured by clonogenic assay. *p < 0.05 compared with shCtl-treated cells. **G** Quantitative analysis the relative expression of *LSD1* and *ERK2* (*MAPK1*) proteins in Hep3B and Huh7 CSCs in response to sh*LSD1*, oe-*ERK2* or in combination by Western blot, relative to *GAPDH*. **H** The LCSC stemness after treatment with sh*LSD1*, oe-*ERK2* or in combination measured by microsphere formation assay. **I**, After 6 Gy X-ray treatment, cell proliferation in the presence of sh*LSD1*, oe-*ERK2* or in combination, as examined by clonogenic assay. *p < 0.05 compared with shCtl + oe-NC-treated cells. #*p* < 0.05 compared with sh*LSD1* + oe-NC-treated cells. Above data were shown as mean ± standard deviation. Results between two groups were compared by unpaired *t* test. The data comparison between multiple groups was performed by one-way ANOVA with Tukey’s post-hoc test. Cellular experiments were repeated in triplicate

We moved to investigate whether the effect of *HOTAIR*-*LSD1* axis on increasing radioresistance was achieved by regulating ERK2 (*MAPK1*). Based on TCGA database, we analyzed the ERK2 (*MAPK1*) expression in liver cancer and its relationship with the overall survival of liver cancer patients, and the results revealed that ERK2 (*MAPK1*) was highly expressed in clinical tissue samples of liver cancer patients (Fig. 4A) and that ERK2 (*MAPK1*) expression was negatively correlated with the overall survival of patients (Fig. 4B, Additional file 2: Figure S3A). Moreover, the results of immunohistochemistry demonstrated that the positive expression of LSD1 and ERK2 (*MAPK1*) was increased in liver cancer tissues (Fig. 4C).

Next, we designed two independent shRNAs targeting ERK2 (*MAPK1*) (sh*ERK2*#1 and sh*ERK2*#2) and transfected them into Hep3B and Huh7 CSCs. The two shRNAs had excellent silencing efficiency, but sh*ERK2*#2 (written as sh*ERK2* in the following text) showed a superior silencing efficiency (Fig. 4D) and was chosen for subsequent experiments. Interestingly, results have revealed that ERK2 (*MAPK1*) activation was critical for maintaining the stemness of stem cells [40]. Here, in the presence of ERK2 (*MAPK1*) silencing, the microsphere formation ability of Hep3B and Huh7 CSCs was reduced (Fig. 4E), revealing the significance of ERK2 (*MAPK1*) in maintenance of LCSCs subsets. Meanwhile, ERK2 (*MAPK1*) silencing accelerated the inhibitory effect of X-ray on the colony formation ability of LCSCs (Fig. 4F). Western blot results revealed that silencing of LSD1 decreased ERK2 (*MAPK1*) expression, which was reversed after further ERK2 (*MAPK1*) overexpression (Fig. 4G, Additional file 2: Figure S3B). In addition, silencing of *LSD1* was found to attenuate the ability of LCSCs to form microspheres, but restored ERK2 (*MAPK1*) expression reversed this result (Fig. 4H). After treatment with X-ray irradiation, *LSD1* silencing markedly restricted the proliferation of LCSCs, and ERK2 (*MAPK1*) overexpression reversed above effects (Fig. 4I). Overall, these results suggested that *LSD1* could upregulate ERK2 (*MAPK1*) expression to maintain the stemness of LCSCs and enhance their radioresistance.

### JMJD6 maintains the stemness of LCSCs and enhances their radioresistance by promoting HOTAIR expression

As previously reported, *JMJD6* and *BRD4* could be recruited to the *HOTAIR* promoter region and further increase *HOTAIR* expression [32, 33]. Besides, JMJD6 and BRD4 could form a complex to modulate gene expression [34], so we speculated that the JMJD6 may form a complex with BRD4 to regulate the expression of *HOTAIR*.

**Fig. 5:**
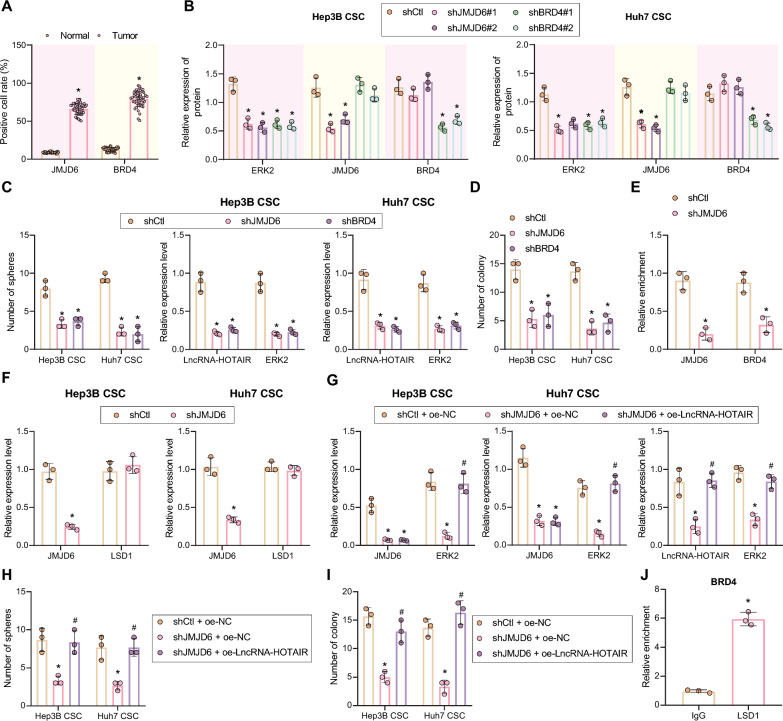
*JMJD6* enhances *HOTAIR* expression to maintain the stemness of LCSCs and promotes their radioresistance. **A** Quantitative analysis of JMJD6 and BRD4 expression in liver cancer and adjacent normal tissues measured by immunohistochemistry. *p < 0.05 compared with adjacent normal tissues. **B** The expression of JMJD6, BRD4 and ERK2 (*MAPK1*) in Hep3B and Huh7 CSCs with *JMJD6* silencing or *BRD4* silencing detected by Western blot, relative to GAPDH. *p < 0.05 compared with shCtl-treated cells. **C** The stemness of LCSCs with *JMJD6* silencing or *BRD4* silencing detected by microsphere formation assay. *p < 0.05 compared with shCtl-treated cells. **D** After exposure to X-ray, the cell colony formation in the presence of sh*JMJD6* or sh*BRD4*, *p < 0.05 compared with shCtl-treated cells. **E** The enrichment of *JMJD6* and *BRD4* in the promoter region of *HOTAIR* after *JMJD6* silencing, as examined by ChIP assay, relative to Input. The relative enrichment on the Y-axis represents *JMJD6* or *BRD4*/IgG. *p < 0.05 compared with shCtl-treated cells. **F** Detection of expression of JMJD6 and LSD1 in cells following silencing of *JMJD6*, *p < 0.05 compared with shCtl-treated cells. **G** The expression of ERK2 (*MAPK1*) and *JMJD6* after *JMJD6* depletion or in combination with *HOTAIR* overexpression, as examined by Western blot and RT-qPCR. *p < 0.05 compared with shCtl + oe-NC-treated cells. #*p* < 0.05 compared with sh*JMJD6* + oe-NC-treated cells. **H** The microsphere formation assay was adopted to measure LCSC stemness after *JMJD6* depletion or in combination with *HOTAIR* overexpression. *p < 0.05 compared with shCtl + oe-NC-treated cells. #*p* < 0.05 compared with sh*JMJD6* + oe-NC-treated cells. **I** Cell colony formation after *JMJD6* depletion or in combination with *HOTAIR* overexpression following 6 Gy X-ray treatment examined by clonogenic assay. *p < 0.05 compared with shCtl + oe-NC-treated cells. #*p* < 0.05 compared with sh*JMJD6* + oe-NC-treated cells. J, RIP assay validated the interaction of JMJD6/BRD4 complex with LSD1 (relative enrichment in Y-axis represents IP-LSD1/IgG, * p < 0.05 compared with IgG group. Measurement data were expressed as mean ± standard deviation. Results between two groups were compared by unpaired *t* test. The data comparison between multiple groups was performed by one-way ANOVA with Tukey’s post-hoc test. Cellular experiments were repeated three times

For verification, we first detected the expression of *JMJD6* and *BRD4* in liver cancer tissues and adjacent normal tissues by RT-qPCR. As shown in Fig. 5A, *BRD4* and *JMJD6* were highly expressed in liver cancer tissues. Additionally, the expression of *HOTAIR* and ERK2 (*MAPK1*) was diminished in Hep3B and Huh7 CSCs after *JMJD6* silencing or *BRD4* silencing (Fig. 5B, Additional file 2: Figure S4). Next, we analyzed the effect of *JMJD6* or *BRD4* on microsphere forming ability of LCSCs, and the results uncovered that *JMJD6* silencing or *BRD4* silencing reduced microsphere forming ability (Fig. 5C, D). Further, the results of ChIP assay on LCSCs displayed that *JMJD6* silencing reduced the enrichment of *JMJD6* and *BRD4* in the *HOTAIR* promoter region (Fig. 5E). The above results indicated that the JMJD6–BRD4 complex enhanced *HOTAIR* expression by binding to its promoter region.

We first detected the expression of LSD1 in JMJD6-silenced cells, and the results depicted that there was no significant change in LSD1 expression after JMJD6 silencing (Fig. 5F, Additional file 2: Figure S3C), suggesting that although JMJD6 regulated HOTAIR transcription by binding to its promoter region, and regulated the binding of LSD1 in the ERK2 promoter region and the enrichment of H3K9me2 through HOTAIR, which affects the radioresistance of LCSCs, but *HOTAIR* did not affect the expression of LSD1 in cells. Next, we overexpressed *HOTAIR* in *JMJD6* silencing cells to the base level, and illustrated that the expression of ERK2 (*MAPK1*) was impeded in response to *JMJD6* depletion, while *HOTAIR* overexpression negated this effect (Fig. 5G). Consistently, an obvious decline in cell microsphere formation and colony formation ability caused by *JMJD6* silencing could be reversed by further overexpression of *HOTAIR* (Fig. 5H, I). It has been reported that JMJD6 can catalyze the hydroxylation of lysine residues, and 19 hydroxylation sites located in the lysine-rich region on BRD4 can be modified by it [41]. As proved by RIP, BRD4 could interact with LSD1 (Fig. 5J). In summary, the JMJD6/BRD4 complex exerted a direct effect on LSD1.

These data revealed that the JMJD6–BRD4 complex could bind to the *HOTAIR* promoter to elevate *HOTAIR* expression and promote ERK2 (*MAPK1*) expression, maintaining the stemness of LCSCs and enhancing cell radioresistance.

### The role of JMJD6 in promoting radioresistance of LCSCs depends on the transcriptional regulation of ERK2 (MAPK1) by HOTAIR-LSD1 axis

**Fig. 6:**
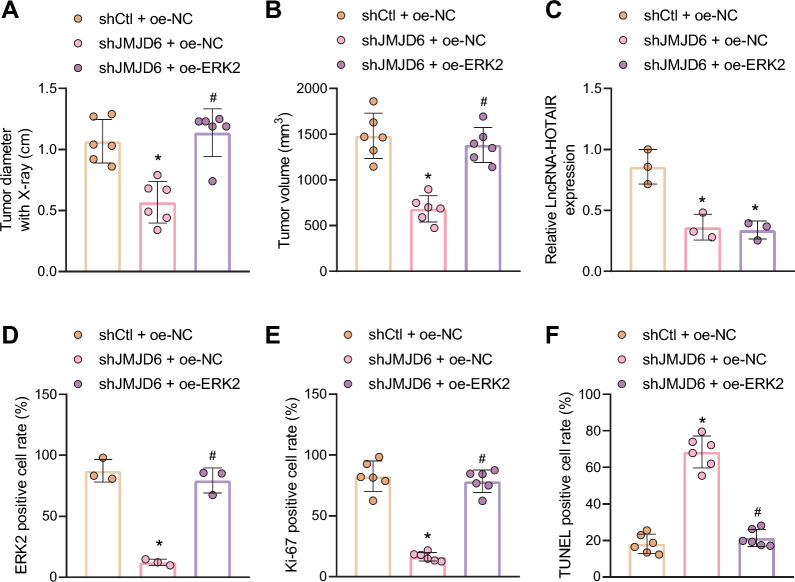
*JMJD6* promotes radioresistance of LCSCs by regulating the *HOTAIR-LSD1-ERK2* axis. **A** The diameter of xenograft tumors of nude mice in response to *JMJD6* silencing alone or in combination with ERK2 (*MAPK1*) overexpression (n = 6). **B** The weight of xenografted tumors in nude mice in response to *JMJD6* silencing alone or in combination with ERK2 (*MAPK1*) overexpression (n = 6). **C** The expression of *HOTAIR* in xenografted tumor tissues of nude mice in response to *JMJD6* silencing alone or in combination with ERK2 (*MAPK1*) overexpression determined by RT-qPCR, normalized to *GAPDH* (n = 6). **D** The ERK2 (*MAPK1*) positive cells in xenograft tumor tissues of nude mice in response to *JMJD6* silencing alone or in combination with ERK2 (*MAPK1*) overexpression, as detected by immunohistochemistry (n = 6). **E** Cell proliferation in xenografted tumors of mice in response to *JMJD6* silencing alone or in combination with ERK2 (*MAPK1*) overexpression, as detected by Ki-67 staining (n = 6). **F** Cell apoptosis in xenografted tumors of mice in response to *JMJD6* silencing alone or in combination with ERK2 (*MAPK1*) overexpression, as detected by TUNEL staining (n = 6). *p < 0.05 compared with shCtl + oe-NC-treated mice. #*p* < 0.05 compared with sh*JMJD6* + oe-NC-treated mice. Measurement data were represented as mean ± standard deviation. The data comparison between multiple groups was performed by one-way ANOVA with Tukey’s post-hoc test

Further, through cellular tumorigenicity in nude mice, we explored whether *JMJD6*-mediated radioresistance in liver cancer cells was related to the regulation of ERK2 (*MAPK1*) by the *HOTAIR*-*LSD1* axis. Hep3B CSCs with *JMJD6* silencing or in combination with ERK2 (*MAPK1*) overexpression were subcutaneously injected to nude mice, followed by exposure to X-ray radiation. After treatment with X-ray, the tumor-forming ability of cells in nude mice was markedly diminished in response to *JMJD6* depletion, while further ERK2 (*MAPK1*) overexpression increased the tumor volume and recovered the resistance of cells to radiation (Fig. 6A, B).

Moreover, in the tumor tissues of mice after *JMJD6* silencing, the expression of *HOTAIR* was restricted, while further overexpression of ERK2 (*MAPK1*) did not affect the expression of *HOTAIR* (Fig. 6C). The results of immunohistochemistry showed that *JMJD6* depletion alone could impede the positive expression of ERK2 (*MAPK1*), which was negated after both *JMJD6* depletion and ERK2 (*MAPK1*) overexpression (Fig. 6D). More importantly, the results of Ki-67 staining and TUNEL assay indicated that silencing of *JMJD6* inhibited the tumor radioresistance and enhanced cell apoptosis. However, ERK2 (*MAPK1*) overexpression negated the impact of *JMJD6* silencing (Fig. 6E, F). To sum up, the promoting effect of *JMJD6* on radioresistance of LCSCs may rely on the transcriptional regulation of ERK2 (*MAPK1*) by *HOTAIR*-*LSD1* axis.

### JMJD6 inhibitor SKLB325 reduces radioresistance of LCSCs by repressing LCSC stemness

**Fig. 7:**
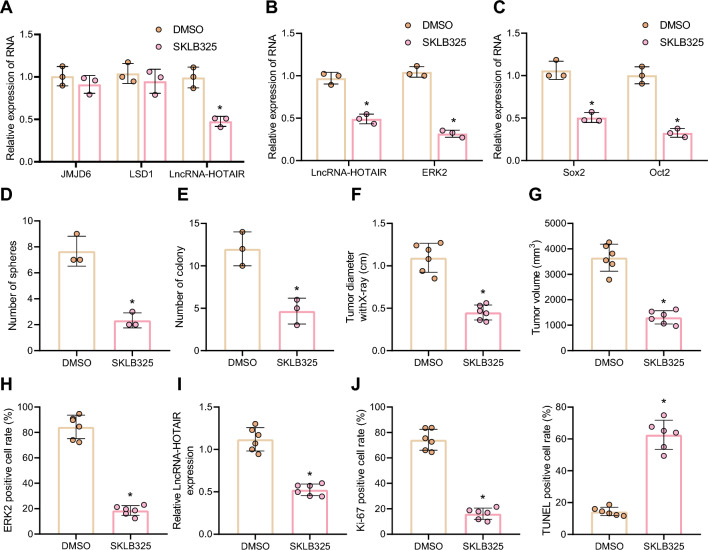
JMJD6 inhibitor SKLB325 represses the LCSC stemness and relieves cell radioresistance. **A** The expression of *HOTAIR*, *JMJD6* and *LSD1* in Hep3B and Huh7 CSCs in response to JMJD6 inhibitor SKLB325 determined by RT-qPCR, normalized to *GAPDH*. **B** The expression of *HOTAIR* and *MAPK1* in Hep3B and Huh7 CSCs in response to JMJD6 inhibitor SKLB325 determined by RT-qPCR, normalized to *GAPDH*. **C** The expression of *Sox2* and *Oct2* in Hep3B and Huh7 CSCs in response to JMJD6 inhibitor SKLB325 determined by RT-qPCR, normalized to *GAPDH*. **D** The difference of cell stemness after SKLB325 treatment, as detected by microsphere formation assay. **E** The impact of SKLB325 on colony formation ability in response to SKLB325 treatment, as detected by clonogenic assay. **F** The diameter of xenografts tumors in mice with SKLB325 treatment. **G** The tumor volume of xenografted tumors. H, ERK2 (*MAPK1*) expression in the tumor tissues of mice in response to JMJD6 inhibitor SKLB325 measured by immunohistochemistry. **I** The expression of *HOTAIR* in the tumor tissues of mice in response to JMJD6 inhibitor SKLB325 determined by RT-qPCR, normalized to *GAPDH*. **J** Ki-67 staining and TUNEL staining were adopted to examine cell proliferation and apoptosis in the tumor tissues of mice in response to JMJD6 inhibitor SKLB325. *p < 0.05 compared with DMSO-treated cells or mice. Experimental data were shown as mean ± standard deviation. Results between two groups were compared by unpaired *t* test

The first JMJD6 inhibitor SKLB325 and its role in cancer were reported in 2019, and until now, few studies focused on the application of *JMJD6* inhibitor in cancer [36]. Therefore, on the basis of above study, we further investigated the effect of *JMJD6* inhibitor on cancer. As presented in Fig. 7A–C, SKLB325 restricted the expression of *HOTAIR*, *MAPK1* and stem cell marker genes *Sox2* and *Oct2* in Hep3B and Huh7 CSCs. Besides, after treatment with SKLB325, the percentage microsphere formation in LCSCs was reduced (Fig. 7D), indicating that SKLB325 could effectively restrict the stemness of LCSCs. Meanwhile, SKLB325 accelerated the inhibitory effect of X-ray on the colony formation ability of LCSCs (Fig. 7E). In the nude mice with xenograft tumors, the tumor-forming ability of SKLB325-treated LCSCs was markedly reduced after radiation (Fig. 7F, G). In addition, after SKLB325 treatment, the expression of *HOTAIR* and ERK2 (*MAPK1*) was diminished in the tumor tissues (Fig. 7H, I), accompanied with slow proliferation and accelerating apoptosis of tumor cells (Fig. 7J). Collectively, JMJD6 inhibitor SKLB325 could effectively attenuate radioresistance of liver cancer cells by reducing LCSC stemness in vivo and in vitro.

## Discussion

**Fig. 8:**
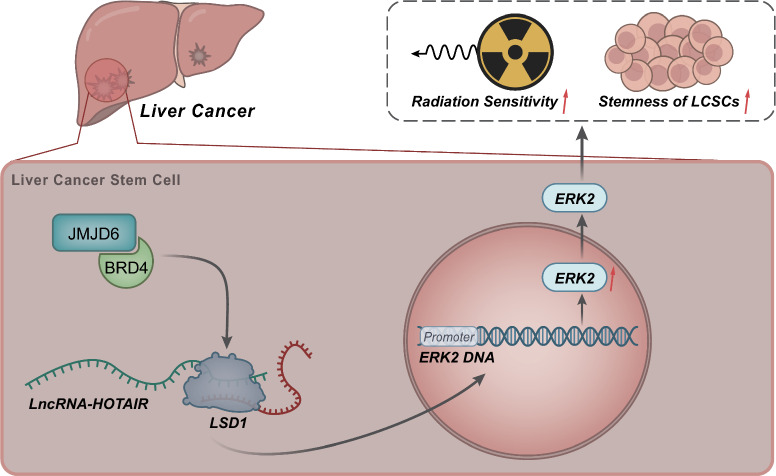
Mechanism graph of the regulatory network. The JMJD6–BRD4 complex elevates the expression of ERK2 (*MAPK1*) through the *HOTAIR*-*LSD1* axis, thereby maintaining the stemness of LCSCs and enhancing their radioresistance

Radiotherapy is a more and more important tool in for the treatment of primary and metastatic liver tumors, and low-dose radiation could restrict unresectable primary tumors or liver metastases [42]. However, although the efficacy of this therapy has been improved in recent years, ultimately radiation delivery was impeded by radioresistance, high toxicity, and a relatively high occurrence of liver failure [8, 43]. According to bioinformatics analysis, we uncovered that *HOTAIR* was expressed at high levels in liver cancer tissues, and its expression has also been reported to be closely associated with the development and poor prognosis of liver cancer [24, 25]. But few studies focus on the effect of *HOTAIR* on the radioresistance of liver cancer cells, as well as its related molecular mechanisms. In the present study, we demonstrate that *HOTAIR* maintains the LCSC stemness and further enhances their radioresistance via the *JMJD6*-*BRD4*-*HOTAIR*-*LSD1*-ERK2 (*MAPK1*) axis (Fig. 8).

*HOTAIR* plays a pivotal role in the pathogenesis of several cancers, the ectopic expression of which is involved in drug resistance, enhancing cell viability and metastasis through various signaling pathways in tumors [44]. Hence, the properties of *HOTAIR* have aroused interest of using *HOTAIR* as a potential therapeutic target or biomarker in cancer biology [45, 46]. In addition, the increased expression of *HOTAIR* is highly linked to cell metastasis, drug tolerance and unfavorable overall survival of patients [47]. In the present study, we also implied that *HOTAIR* enhanced the radioresistance by maintaining the stemness of LCSCs.

As highly reported, *HOTAIR* could act as an *LSD1* scaffold molecule to modulate the expression of genes [30, 48, 49]. Moreover, *LSD1* expression has been found to be enhanced in several cancers and this enhancement can facilitate the proliferation, drug resistance and transformation of cancer cells in vitro and in vivo [50–52]. Liu et al*.* has uncovered that *LSD1* is highly expressed in LCSCs and closely linked to liver cancer progression [33], which is consistent with our results in Fig. 2. Further, Li et al*.* have illustrated that *LSD1* up-regulates the expression of ERK [38]. ERK plays a significant role in the intracellular regulating network, and the MAPK–ERK cascades can be activated by various stimuli, including the extracellular matrix and growth factors [53–55]. Besides, CD133^+^ LCSCs display a marked increase in the MAPK pathway activation, as supported by phosphorylated ERK level [56]. Meanwhile, ERK signal has been proposed to be implicated in the progression of various human tumors by mediating the expression of genes, such as cyclin D1 [57] and Bax [58]. In this study, we demonstrated that *HOTAIR* maintained the LCSC stemness and enhance its radioresistance by regulating *LSD1* and further promoting ERK2 (*MAPK1*) expression. Consistent with our results, Piao et al*.* also uncovered that abnormal activation of ERK could increase the radioresistance of liver cancer cells [39]. In addition, overexpression of *HOTAIR* has been reported to accelerate dyskinesia and facilitate dopaminergic neuron apoptosis in a Parkinson’s disease mouse model via activation of the ERK1/2 axis [59]. Another study has shown that *HOTAIR* depletion can promote apoptosis of lymphoma cells via inactivation of the ERK signaling pathway [60].

*JMJD6*, an epigenetic mediator of multilevel activities [35], has been unveiled to promote the expression of *HOTAIR* [32]. Additionally, *JMJD6* has been indicated to participate in the development of multiple cancers, including liver cancer [61–63], and the enzymatic activity of *JMJD6* was pivotal for cancer progression [64, 65]. Thus, inhibiting *JMJD6* activity was recognized as a promising solution for the treatment of *JMJD6*-involved cancers [66]. Recently, Zheng et al*.* found a *JMJD6* inhibitor, SKLB325, diminished intraperitoneal tumor weight and prolong the overall survival of tumor-bearing nude mice, implying that SKLB325 might be a potential candidate drug for cancer management [36]. In the current study, our results also showed that SKLB325 restricted the stemness of LCSCs and attenuated their radioresistance in vivo and in vitro by mediating *HOTAIR* expression.

Taken together, our experimental results showed that *HOTAIR* impeded the radiosensitivity of liver cancer cells by maintaining its stemness. Besides, the effect of *HOTAIR* on LCSCs was achieved by recruiting *LSD1* to enhance ERK2 (*MAPK1*) expression. Further, the expression of *HOTAIR* was regulated by JMJD6–BRD4 complex, and the JMJD6 inhibitor contributed to the radiosensitivity of liver cancer cells by suppressing the expression of *HOTAIR* and ERK2 (*MAPK1*). Based on the data of our study, we demonstrated that JMJD6–BRD4 complex regulated the LCSC irradiation tolerance via the *HOTAIR*-*LSD1*-*ERK2 *(*MAPK1*) axis, which provided promising targets or biomarkers for the management of radioresistance in liver cancer.

### Supplementary Information

**Additional file 1: Table S1.** The sequences of shRNAs. **Table S2.** The primer sequences of RT-qPCR. **Table S3.** LncRNAs associated with liver cancer in the lncDieseas database.

**Additional file 2: Figure S1.** A heat map of the expression of differentially expressed genes in CD13^+^CD133^+^ or negative liver cancer cell subsets in the RNA-seq data. **Figure S2.** Representative images of microsphere formation and colony formation assays. **Figure S3.** Representative images of Western blots. **Figure S4.** Quantitative analysis of the *HOTAIR* expression in Hep3B and Huh7 CSCs with *JMJD6* silencing or *BRD4* silencing, relative to *GAPDH*.

## Data Availability

The datasets generated and/or analyzed during the current study are available from the corresponding author on reasonable request.

## Abbreviations

LCSCs: Liver cancer stem cells
*HOTAIR*: HOX transcript antisense RNA
*LSD1*: Lysine-specific demethylase 1
*JMJD6*: Jumonji Domain Containing 6
*BRD4*: Bromodomain-containing protein 4
HRP: Horseradish peroxidase
RT-qPCR: Reverse transcription quantitative polymerase chain reaction
sh: Short hairpin RNA
oe: Overexpression
DMEM: Dulbecco’s modified Eagle’s medium
NC: Negative control
FBS: Fetal bovine serum
EDTA: Ethylenediaminetetraacetic acid
RIP: RNA immunoprecipitation
DAB: 3,3ʹ-Diaminobenzidine tetrahydrochloride
IgG: Immunoglobulin G
TUNEL: Terminal deoxynucleotidyl transferase-mediated dUTP-biotin nick end labeling
GAPDH: Glyceraldehyde-3-phosphate dehydrogenase
ANOVA: Analysis of variance
