# Supplementary material for: JMJD6–BRD4 complex stimulates lncRNA HOTAIR transcription by binding to the promoter region of HOTAIR and induces radioresistance in liver cancer stem cells
Source: J Transl Med. 2023 Oct 25;21:752. doi: 10.1186/s12967-023-04394-y (PMC10599021; doi:10.1186/s12967-023-04394-y)
Supplement: Supplementary file 1 — Additional file 1: Table S1. The sequences of shRNAs. Table S2. The primer sequences of RT-qPCR. Table S3. LncRNAs associated with liver cancer in the lncDieseas database. [file 12967_2023_4394_MOESM1_ESM.docx]

**Table S1** The sequences of shRNAs.

| Target | Sequence (5'-3') |
| --- | --- |
| shCtl | GGGTGAACTCACGTCAGAA |
| shHOTAIR | GCCTTTGCTTCGTGCTGA |
| shJMJD6#1 | GAGGGAACCAGCAAGACGA |
| shJMJD6#2 | GTGTGGTGAGGATAACGAT |
| shLSD1#1 | GGACTTCAAGACGACAGTTCT |
| shLSD1#2 | GCCACATTTCGCAAAGGAAAC |
| shBRD4#1 | GCCAAATGTCTACACAGTATA |
| shBRD4#2 | CAGTGACAGTTCGACTGATGA |
| shERK2#1 | AGCCAGGATACAGATCTTAAA |
| shERK2#2 | CCCATATCTGGAGCAGTATTA |

Note: shCtl, shRNA control; shlncRNA HOTAIR, shRNA targeting lncRNA HOTAIR; shJMJD6, shRNA targeting JMJD6; shBRD4, shRNA targeting BRD4; shERK22, shRNA targeting ERK22.

**Table S2** The primer sequences of RT-qPCR.

| Gene | Sequence (5’-3’) |
| --- | --- |
| *HOTAIR* | F: CAGTGGGGAACTCTGACTCG |
|  | R: GTGCCTGGTGCTCTCTTACC |
| *JMJD6* | F: TTGGACCCGGCACAACTACTA |
|  | R: TCTGCCCTTTCCACGTTATCC |
| *LSD1* | F: TGACCGGATGACTTCTCAAGA |
|  | R: GTTGGAGAGTAGCCTCAAATGTC |
| *SOX2* | F: GCCGAGTGGAAACTTTTGTCG |
|  | R: GGCAGCGTGTACTTATCCTTCT |
| *OCT2* | F: GGCCCCAGTACAAAGATCAA |
|  | R: GTTTGCTGAGGTAGCTGGAA |
| *MAPK1* | F: TACACCAACCTCTCGTACATCG |
|  | R: CATGTCTGAAGCGCAGTAAGATT |
| *GAPDH* | F: GTGGACCTGACCCTGCCGTCT |
|  | R: GGAGGAGTGGGTGTCGCTGT |

Note: F, forward; R, reverse; HOTAIR, HOX transcript antisense RNA; JMJD6, jumonji domain containing 6; LSD1, lysine demethylase 1; SOX2, SRY-box transcription factor 2; MAPK1, mitogen-activated protein kinase 1; GAPDH, glyceraldehyde-3-phosphate dehydrogenase.

**Table S3** LncRNAs associated with liver cancer in the lncDieseas database.

| LncRNA name | Disease name | Dysfunction type | Description | Chr | Start | End | Strand | Species | Alias | Genbank | Sequence | Reference |
| --- | --- | --- | --- | --- | --- | --- | --- | --- | --- | --- | --- | --- |
| H19 | liver cancer | Epigenetics | H19 ICR showed loss-of-imprinting in two steps and allelic histone marker signature during tumorigenesis showed similarity with ES cells. | chr11 | 1995176 | 2001466 | - | Human | ASM; ASM1; BWS; D11S813E; LINC00008; NCRNA00008; WT2 | NR_131223 | Gene / RNA | 21163252 |
| H19 | liver cancer | Regulation | Control of imprinting breast, cervix, oesophagus prostate, endometrial, colon | chr11 | 1995176 | 2001466 | - | Human | ASM; ASM1; BWS; D11S813E; LINC00008; NCRNA00008; WT2 | NR_131223 | Gene / RNA | 24499465 |
| H19 | liver cancer | Regulation | Control of imprinting. Containing miRNA miR-675. | chr11 | 1995176 | 2001466 | - | Human | ASM; ASM1; BWS; D11S813E; LINC00008; NCRNA00008; WT2 | NR_131223 | Gene / RNA | 22996375 |
| HOTAIR | liver cancer | Expression | In approximately one-quarter of human breast cancers, HOTAIR is highly induced, while its elevated levels are also predictive of metastasis and disease progression in other cancers, such as colon, colorectal, gastrointestinal, pancreatic and liver cancer. | chr12 | 53962308 | 53974956 | - | Human | HOXAS; HOXC-AS4; HOXC11-AS1; NCRNA00072 | NR_003716 | Gene / RNA | 24667321 |
| HULC | liver cancer | N/A | PCGEM1, PCA3 (prostate cancer antigen 3, known also as DD3, differential display code 3) and PCNCR1 (prostate cancer ncRNA 4) are involved in prostate cancer, while HULC (highly up-regulated in liver cancer) is involved with liver cancer. | chr6 | 8652209 | 8653846 | + | Human | HCCAT1; LINC00078; NCRNA00078 | NR_004855 | Gene / RNA | 24667321 |
| HULC | liver cancer | Expression | The highly upregulated lncRNA HULC in liver cancer was found in the blood of HCC patients, promising a potential biomarker. | chr6 | 8652209 | 8653846 | + | Human | HCCAT1; LINC00078; NCRNA00078 | NR_004855 | Gene / RNA | 24531795 |
| LncRNA-LALR1 | liver cancer | Regulation | LncRNA-LALR1 accelerates hepatocyte proliferation during liver regeneration by activating Wnt/β-Catenin signaling | N/A | N/A | N/A | N/A | Human | N/A | N/A | Gene / RNA | 23483581 |
| MALAT1 | liver cancer | Regulation | Sequesters SR splicing factors to regulate alternative splicing. | chr11 | 65497679 | 65504494 | + | Human | HCN; LINC00047; NCRNA00047; NEAT2; PRO2853 | NR_002819 | Gene / RNA | 22996375 |
| MALAT1 | liver cancer | Regulation | Mutual inhibition between YAP and SRSF1 maintains long non-coding RNA, Malat1-induced tumourigenesis in liver cancer. | chr11 | 65497679 | 65504494 | + | Human | HCN; LINC00047; NCRNA00047; NEAT2; PRO2853 | NR_002819 | Gene / RNA | 24468535 |
| lncCAMTA1 | liver cancer | Regulation | Long Noncoding RNA lncCAMTA1 Promotes Proliferation and Cancer Stem Cell-Like Properties of Liver Cancer by Inhibiting CAMTA1. | N/A | N/A | N/A | N/A | Human | N/A | N/A | Gene / RNA | 27669232 |
| UCA1 | liver cancer | Regulation | Double mutant P53 (N340Q/L344R) promotes hepatocarcinogenesis through upregulation of Pim1 mediated by PKM2 and LncRNA CUDR. | chr19 | 15828947 | 15836321 | + | Human | CUDR; LINC00178; NCRNA00178; UCAT1; onco-lncRNA-36 | NR_015379 | Gene / RNA | 27167190 |
| DILC | liver cancer | Expression | Lnc-DILC could be not only a potential prognostic biomarker, but also a possible therapeutic target against LCSCs. | N/A | N/A | N/A | N/A | Human | N/A | N/A | Gene / RNA | 26812074 |
| HULC | liver cancer | Regulation | HULC promotes tumor angiogenesis in liver cancer through miR 107/E2F1/SPHK1 signaling. | chr6 | 8652209 | 8653846 | + | Human | HCCAT1; LINC00078; NCRNA00078 | NR_004855 | Gene / RNA | 26540633 |
| UCA1 | liver cancer | Regulation | Long noncoding RNA CUDR will help in the development of new liver cancer therapeutic and diagnostic approaches. | chr19 | 15828947 | 15836321 | + | Human | CUDR; LINC00178; NCRNA00178; UCAT1; onco-lncRNA-36 | NR_015379 | Gene / RNA | 26513297 |
| H19 | liver cancer | Regulation | miR675 upregulates long noncoding RNA H19 through activating EGR1 in human liver cancer. | chr11 | 1995176 | 2001466 | - | Human | ASM; ASM1; BWS; D11S813E; LINC00008; NCRNA00008; WT2 | NR_131223 | Gene / RNA | 26376677 |
| HOTAIR | liver cancer | Regulation | LncRNA HOTAIR promotes human liver cancer stem cell malignant growth through downregulation of SETD2. | chr12 | 53962308 | 53974956 | - | Human | HOXAS; HOXC-AS4; HOXC11-AS1; NCRNA00072 | NR_003716 | Gene / RNA | 26172293 |
| HOTAIR | liver cancer | N/A | PLK1 and HOTAIR Accelerate Proteasomal Degradation of SUZ12 and ZNF198 during Hepatitis B Virus-Induced Liver Carcinogenesis. | chr12 | 53962308 | 53974956 | - | Human | HOXAS; HOXC-AS4; HOXC11-AS1; NCRNA00072 | NR_003716 | Gene / RNA | 25855382 |
| lncTCF7 | liver cancer | Regulation | LncTCF7-mediated Wnt signaling primes liver CSC self-renewal and tumor propagation. | N/A | N/A | N/A | N/A | Human | N/A | N/A | Gene / RNA | 25842979 |
| HULC | liver cancer | Regulation | HULC contributes to the perturbations in circadian rhythm of hepatoma cells. | chr6 | 8652209 | 8653846 | + | Human | HCCAT1; LINC00078; NCRNA00078 | NR_004855 | Gene / RNA | 25622901 |
| MT1DP | liver cancer | Regulation | Tumor suppressor long non-coding RNA, MT1DP is negatively regulated by YAP and Runx2 to inhibit FoxA1 in liver cancer cells | chr16 | 56643705 | 56644786 | + | Human | MT1DP | ENST00000463480 | Gene / RNA | 25261601 |
| MIR7-3HG | liver cancer | Expression | Reduced expression of uc002mbe.2 may be associated with liver carcinogenesis | chr19 | 4769105 | 4772556 | + | Human | C19orf30; Huh7; LINC00306; NCRNA00306; PGSF1; uc002mbe.2 | NR_027148 | Gene / RNA | 23643933 |
