# Supplementary material for: JMJD6–BRD4 complex stimulates lncRNA HOTAIR transcription by binding to the promoter region of HOTAIR and induces radioresistance in liver cancer stem cells
Source: J Transl Med. 2023 Oct 25;21:752. doi: 10.1186/s12967-023-04394-y (PMC10599021; doi:10.1186/s12967-023-04394-y)

**Figure S1** A heat map of the expression of differentially expressed genes in CD13^+^CD133^+^ or negative liver cancer cell subsets in the RNA-seq data.

**
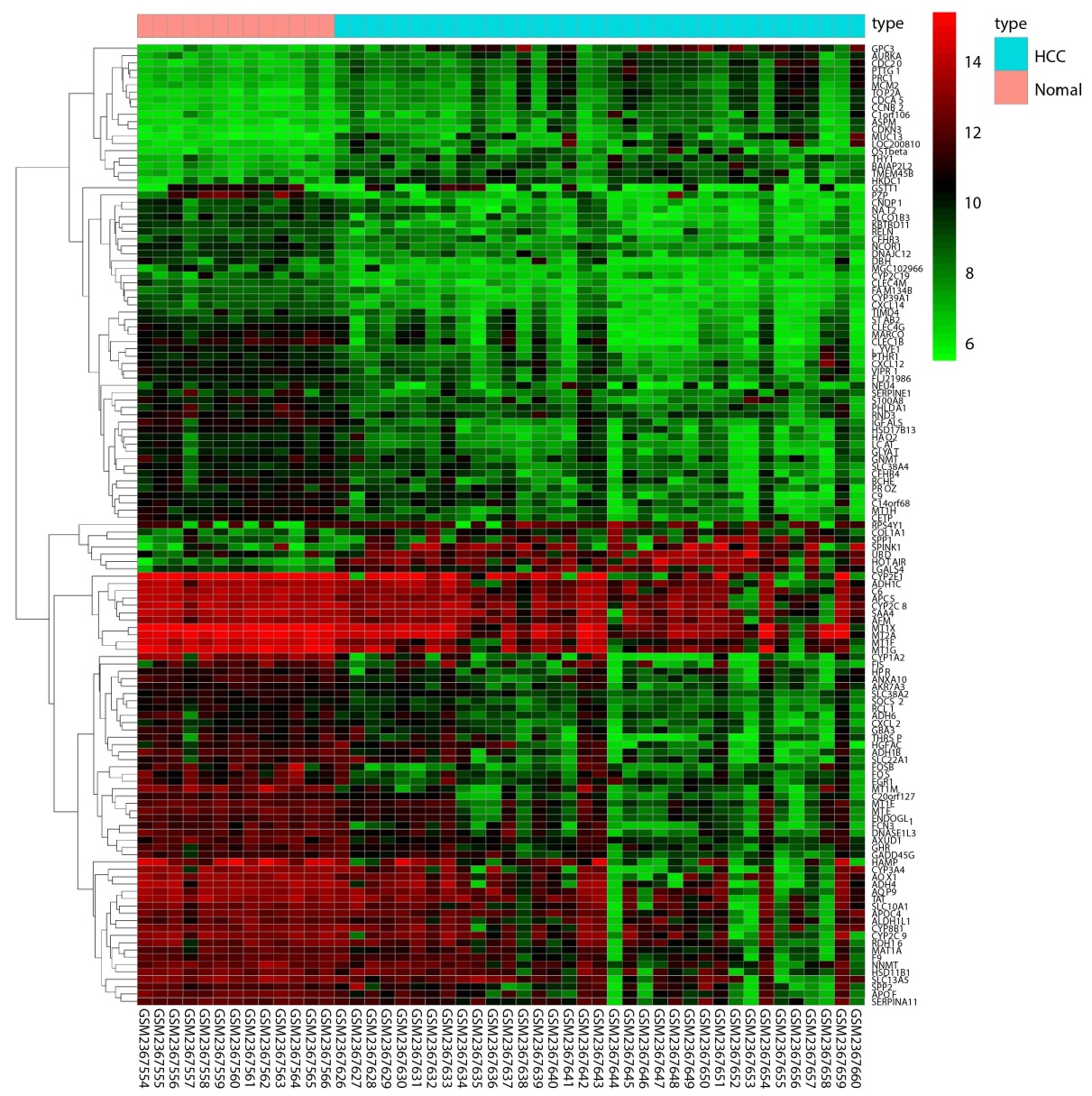
**

**Figure S2** Representative images of microsphere formation and colony formation assays. A, Representative images of Figure 1F. B, Representative images of Figure 1G. C, Representative images of Figure 2E. D, Representative images of Figure 2F.

**
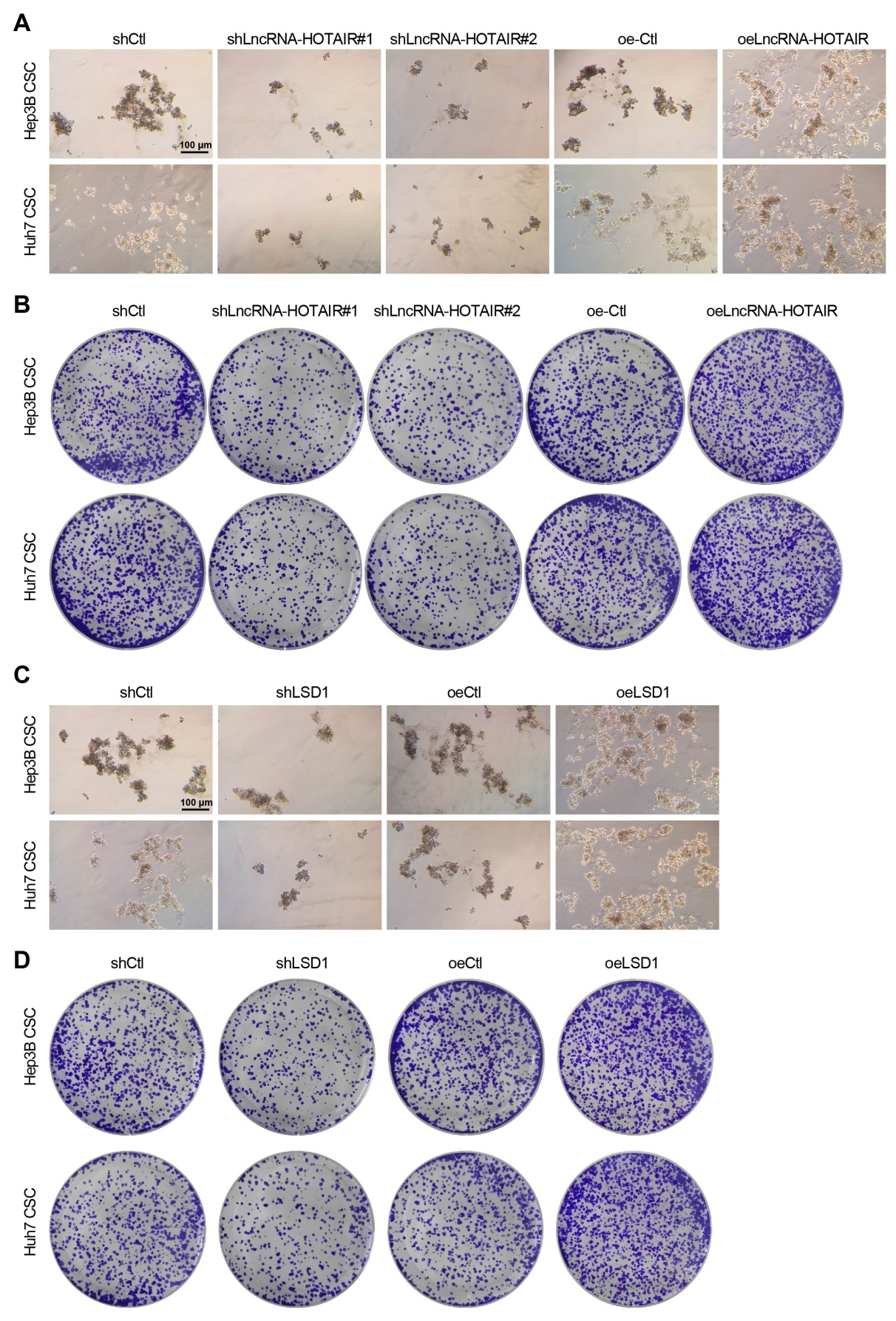
**

**Figure S3** Representative images of Western blots. A, Representative images of Figure 4B, normalized to GAPDH. B, Representative Western blots of LSD1 and ERK2 (*MAPK1*) proteins in Hep3B and Huh7 CSCs in response to sh*LSD1*, oe-*ERK2* or in combination, normalized to GAPDH. C, The expression of JMJD6, ERK2 (*MAPK1*) after *JMJD6* depletion or in combination with *HOTAIR* overexpression.

**
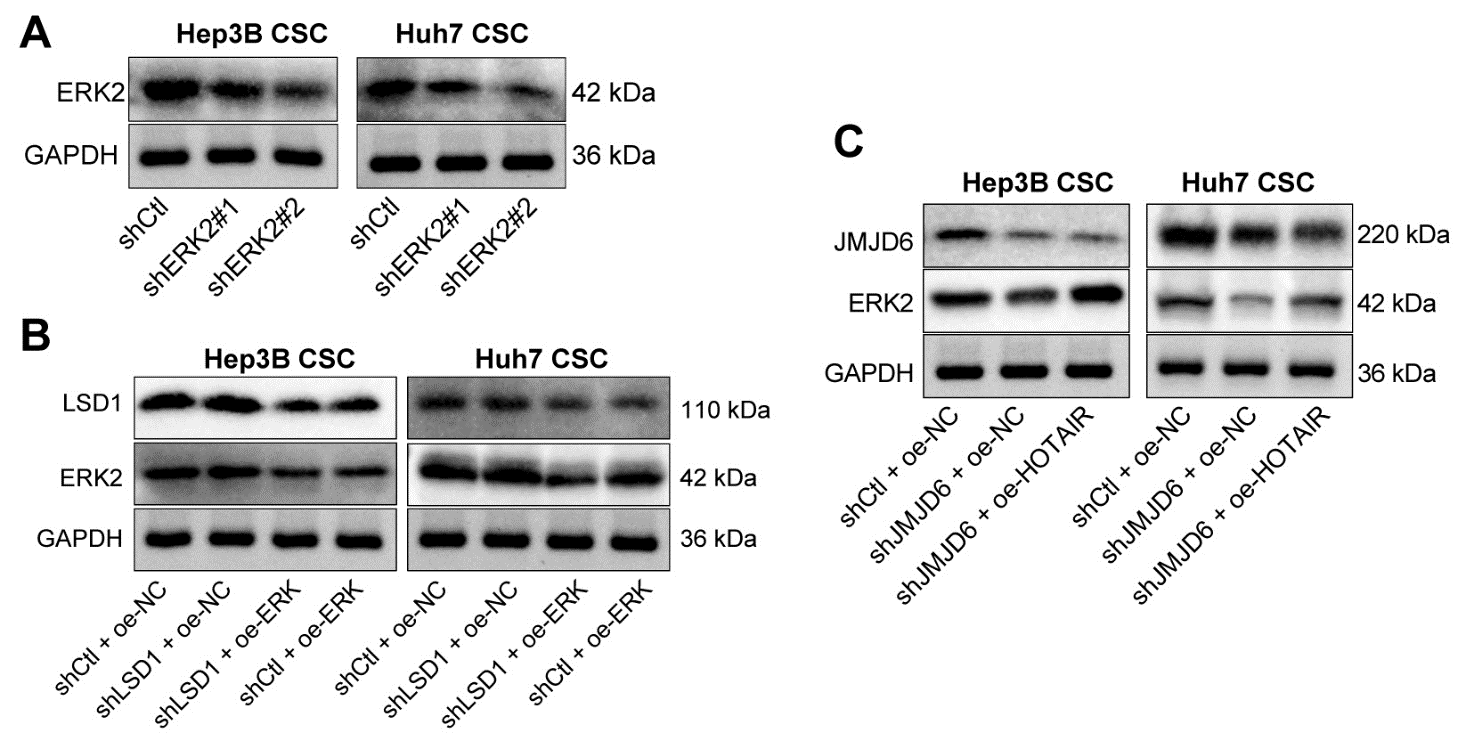
**

**Figure S4** Quantitative analysis of the *HOTAIR* expression in Hep3B and Huh7 CSCs with *JMJD6* silencing or *BRD4* silencing, relative to *GAPDH*.


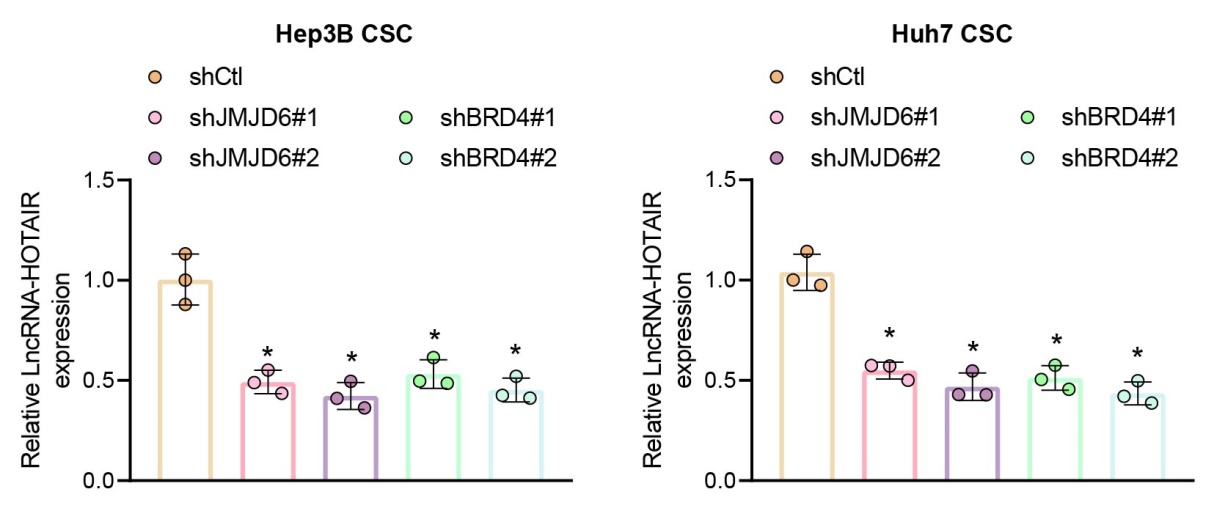

Supplement: Supplementary file 2 — Additional file 2: Figure S1. A heat map of the expression of differentially expressed genes in CD13+CD133+ or negative liver cancer cell subsets in the RNA-seq data. Figure S2. Representative images of microsphere formation and colony formation assays. Figure S3. Representative images of Western blots. Figure S4. Quantitative analysis of the HOTAIR expression in Hep3B and Huh7 CSCs with JMJD6 silencing or BRD4 silencing, relative to GAPDH. [file 12967_2023_4394_MOESM2_ESM.docx]
